# Effect of a modified soft contact lens combined with a noncontact wide-angle viewing system during vitrectomy

**DOI:** 10.1097/MD.0000000000048339

**Published:** 2026-09-04

**Authors:** Jintao Xia, Hongbing Zhang, Lina Cheng, Xiaogang Yang

**Affiliations:** a Department of Ophthalmology, First Affiliated Hospital of Northwest University, Xi’an No. 1 Hospital, Xi’an City, Shaanxi Province, China; b Shaanxi Institute of Ophthalmology, Xi’an City, Shaanxi Province, China.

**Keywords:** noncontact wide-angle viewing system, Ocular Surface Disease Index (OSDI), soft contact lens, surgical field, vitrectomy surgery

## Abstract

**Background:**

To evaluate the efficacy of a modified soft contact lens (MSCL) in maintaining intraoperative visual clarity and protecting the cornea during vitrectomy using a noncontact wide-angle viewing system.

**Methods:**

Patients undergoing pars plana vitrectomy were randomly assigned to a balanced salt solution (BSS) group or an MSCL group. All surgeries were performed with a noncontact wide-angle viewing system. In the BSS group, the cornea was intermittently irrigated with BSS, whereas an MSCL was applied throughout surgery in the MSCL group. Surgical time, vitrectomy duration, intraoperative visual clarity, and frequency of corneal irrigation were recorded. Corneal epithelial edema, fluorescein staining, tear film breakup time, and central corneal thickness were assessed preoperatively and at 1 day, 1 week, and 1 month postoperatively. Ocular Surface Disease Index scores were evaluated preoperatively and at 1 month.

**Results:**

The MSCL group showed significantly better intraoperative visual clarity and corneal hydration than the BSS group (*P* < .05). Surgical time and vitrectomy duration were comparable between groups (*P* > .05). Corneal edema, fluorescein staining, and tear film breakup time at 1 day and 1 week were significantly improved in the MSCL group (*P* < .05), with no differences at 1 month. Ocular Surface Disease Index scores did not differ significantly between groups.

**Conclusion:**

MSCL use effectively protects the cornea and improves intraoperative visualization during vitrectomy without adversely affecting postoperative ocular surface comfort.

## 1. Introduction

With the continuous advancement of medical technology, increasing demands have been placed on the precision and safety of surgical procedures. Minimally invasive vitreoretinal surgery has gained widespread recognition because of its reduced tissue trauma and rapid postoperative recovery.^[1,2]^ Nevertheless, the high level of precision required for these procedures poses substantial challenges in maintaining a consistently clear and stable intraoperative visual field. In vitreoretinal surgery, the quality of visualization directly influences surgical accuracy and safety, making the maintenance of corneal transparency throughout the operation a critical concern for ophthalmic surgeons.

At present, commonly used fundus visualization methods in vitreoretinal surgery include corneal contact lenses and noncontact wide-angle viewing systems.^[3]^ Corneal contact lenses can provide relatively clear visualization; however, their direct contact with the ocular surface may increase the risk of corneal epithelial injury and infection. By contrast, noncontact wide-angle viewing systems have been increasingly adopted in clinical practice because of their ease of use, wide field of view, and avoidance of direct corneal contact.

Despite these advantages, noncontact wide-angle viewing systems still require continuous corneal hydration to maintain optical clarity. During surgery, assistants often need to repeatedly irrigate the cornea with balanced salt solution (BSS) or apply viscoelastic agents to preserve corneal moisture and transparency.^[4]^ Although these measures can temporarily improve visualization, they are labor-intensive, have a limited duration of effectiveness, and may interrupt surgical flow. Moreover, frequent irrigation or viscoelastic application may increase intraoperative complexity and potential risks.

In this context, the present study introduces a modified corneal surface soft lens designed to provide sustained corneal protection and stable visual clarity during vitreoretinal surgery. Based on conventional bandage soft contact lenses (BSCLs),^[5]^ this modified lens is fabricated from a highly biocompatible and transparent material that closely conforms to the corneal surface, thereby reducing dehydration and optical degradation. In addition, its favorable elasticity and stability allow adaptation to different ocular contours and surgical conditions.

To assess the clinical value of this approach, a comparative study was conducted between the modified soft contact lens (MSCL) and conventional BSS corneal irrigation. By evaluating intraoperative visual clarity, postoperative ocular surface parameters, and surgical efficiency, this study aims to clarify the role and potential advantages of the MSCL in vitreoretinal surgery.

## 2. Patients and methods

### 2.1. Ethical approval

This prospective clinical study was approved by the Ethics Committee of Xi’an First Hospital (Approval No. 2022[7]) and conducted in strict accordance with the Declaration of Helsinki. All participants were fully informed about the study objectives, procedures, potential risks, and benefits, and written informed consent was obtained from each patient prior to enrollment.

### 2.2. Patients

A total of 96 eyes from patients who underwent pars plana vitrectomy (PPV) at the Department of Ophthalmology, Xi’an First Hospital, between January 2022 and December 2022 were enrolled. Of these, 44 eyes were excluded: 24 did not meet the inclusion criteria, and 20 declined to participate. The remaining 52 eyes were enrolled and randomly assigned using a computer-generated randomization list without block restriction to either the MSCL group (n = 31) or the BSS group (n = 21). Of the 31 eyes in the MSCL group, 6 were lost to follow-up. The remaining 25 eyes in the MSCL group and 21 eyes in the BSS group completed a 1-month follow-up. Combined phacoemulsification and intraocular lens implantation were performed intraoperatively based on lens opacity, and intraocular lenses were implanted immediately following cataract extraction when indicated. In the MSCL group, a therapeutic corneal BSCL made of silicone hydrogel material (Patent Nos. ZL202010301969.8, ZL202220551842.6, and ZL2022217424233.9) was applied during surgery and sterilized. In the BSS group, BSS (Shenyang Xingqi Pharmaceutical Co., Ltd., Shenyang, Liaoning, China) was used intermittently to maintain corneal hydration. No significant differences were observed in baseline characteristics between groups (*P* > .05, Table 1). Inclusion criteria were patients diagnosed with retinal diseases requiring vitrectomy, corneal endothelial cell density ≥2000 cells/mm^2^, absence of ocular surface disease or significant anterior segment abnormalities, no recent ocular surgery or trauma within 1 month (except for intravitreal anti-vascular endothelial growth factor injection), and suitability for ophthalmic surgery under routine clinical conditions. Exclusion criteria included patient refusal or withdrawal, syncope or intolerance during examination or surgery, incomplete data recording, or other conditions judged by the investigator to affect corneal assessment, such as corneal edema secondary to persistently elevated intraocular pressure.

**Table 1:** Clinical characteristics of patients between MSCL and BSS groups.

Groups	Gender, n		Age (mean ± SD yr)	Mean counts of endothelium
	Male	Female		
MSCL	15	6	55.43 ± 8.34	2633.62 ± 253.64
BSS	18	7	60.52 ± 10.08	2659.28 ± 248.79
Statistic value	0.002*		−1.843^†^	−0.345^†^
*P*	.966		.072	.731

BSS = balanced salt solution, MSCL = modified soft contact lens, SD = standard deviation.

* Chi-square test.

† *t* test.

### 2.3. Surgical procedure

All surgeries were performed by the same experienced vitreoretinal surgeon using standard 3-port PPV with a RESIGHT noncontact wide-angle viewing system (Carl Zeiss Meditec AG, Jena, Thuringia, Germany). Preoperatively, 0.5% levofloxacin eye drops (Zhongshan Wanhan Pharmaceutical Co., Ltd., Zhongshan, Guangdong, China) were administered for infection prophylaxis. The conjunctival sac was disinfected with Anerdian III solution for 60 seconds and rinsed with sterile normal saline. In the MSCL group, a modified corneal bandage lens with a diameter of 10.5 mm was placed on the corneal surface immediately after surgical draping and maintained throughout the procedure. In the BSS group, the cornea was intermittently irrigated with BSS as required to maintain clarity. The duration of vitrectomy was measured from the insertion of the first trocar to the removal of the last trocar. Total operative time and frequency of BSS irrigation were recorded.

### 2.4. Corneal edema classification

Corneal edema was assessed using slit-lamp microscopy and graded on a 5-point scale.^[6]^ Grade 0 represented a transparent cornea without edema, grade 1 indicated localized mist-like edema with smooth endothelium and clear iris details, grade 2 denoted pale gray edema with rough endothelium and blurred iris texture, grade 3 corresponded to diffuse gray-white edema with cracked endothelium and indistinct iris features, and grade 4 represented milky corneal edema with obscured intraocular structures.

### 2.5. Optical clarity of the surgical field

Intraoperative visual clarity was categorized according to previous reports^[7]^ and surgical experience. Grade 1 indicated poor visibility, with the inability to identify retinal vessels or optic disc, requiring corneal epithelial scraping. Grade 2 reflected slightly unclear visualization, allowing basic identification of retinal vessels and optic disc but insufficient for macular procedures. Grade 3 represented clear visualization sufficient for epiretinal membrane peeling, and grade 4 corresponded to excellent visualization enabling delicate macular operations such as epiretinal membrane or internal limiting membrane peeling. Surgical videos were evaluated by the operating surgeon and independently reviewed by 2 senior ophthalmologists.

### 2.6. Tear film breakup time (BUT)

Tear film stability was measured by fluorescein breakup time. Five to ten microliters of fluorescein sodium were instilled into the lower conjunctival sac, and patients were instructed to blink 3 to 4 times. The interval between the last blink and the appearance of the first dark spot on the corneal surface was recorded. A BUT exceeding 10 seconds was considered normal.

### 2.7. Fluorescein staining of the corneal epithelium

Corneal epithelial integrity was evaluated by fluorescein staining under cobalt blue illumination. Each corneal quadrant was scored from 0 to 3, with 0 indicating no staining, 1 indicating 1 to 30 punctate dots, 2 indicating >30 dots with confluent staining, and 3 representing punctate fusion, filaments, or corneal ulcers. The total score ranged from 0 to 12 points.

### 2.8. Measurement of central corneal thickness

Central corneal thickness was measured using anterior segment optical coherence tomography (RTVue XR, Optovue, Inc., Fremont) preoperatively and at 1 day, 1 week, and 1 month postoperatively.^[8–10]^

### 2.9. Questionnaire survey

Ocular surface symptoms were evaluated using the Ocular Surface Disease Index (OSDI) questionnaire before surgery and at 1 month postoperatively.^[11,12]^ The 12-item questionnaire assessed visual symptoms, functional limitations, and environmental triggers. Each item was scored from 0 (none) to 4 (constant occurrence), and the total OSDI score was calculated using the formula: OSDI = ([sum of answered item scores × 100]/[number of answered questions × 4]).

### 2.10. Statistical analysis

Statistical analyses were performed using SPSS 26.0 (International Business Machines Corporation [IBM Corp.], Armonk). Continuous variables with normal distribution, including age, endothelial cell count, and OSDI scores, were compared using the independent-samples *t* test. Non-normally distributed continuous variables, such as operative time, vitrectomy duration, and corneal irrigation frequency, were analyzed using the Mann–Whitney *U* test. Categorical variables, including sex, combined surgery, and surgical field clarity, were compared using the chi-square test, while ordinal variables such as corneal edema grade and fluorescein staining scores were analyzed using the Mann–Whitney *U* test. Intragroup comparisons of fluorescein staining scores were performed using the Wilcoxon signed-rank test. Repeated-measures, two-factor analysis of variance was applied to repeated continuous variables, including BUT and central corneal thickness. A two-sided *P* value < .05 was considered statistically significant.

## 3. Results

### 3.1. Baseline comparison of MSCL with BSS

In the MSCL group, a total of 21 eyes were included, comprising 15 males (71.4%) and 6 females (28.6%). The BSS group consisted of 25 eyes, with 18 males (72.0%) and 7 females (28.0%), demonstrating no significant gender difference (χ^2^ = 0.002, *P* = .966). The mean age in the MSCL group was recorded as 55.43 ± 8.34 years, while it was noted as 60.52 ± 10.08 years in the BSS group (*T* = −1.843, *P* = .072), which did not exhibit any statistically significant variation between them. The preoperative corneal endothelium count was determined to be an average of 2633.62 ± 253.64/mm^2^ in the MSCL group and 2659.28 ± 248.79/mm^2^ in the BSS group (*T* = −0.345, *P* = .732), indicating no statistical significance (Table 1).

### 3.2. Comparison of surgery-related indices between the 2 groups

There was no significant difference in the total operation time and PPV time between the 2 groups by the Mann–Whitney *U* test, but there was a significant difference in the number of times BSS was used for corneal irrigation. The frequency of BSS for corneal flushing was significantly reduced by using the MSCL. The MSCL group had an average of 2.48 ± 0.87 times, and the BSS group had an average of 81.96 ± 24.86 times, and the difference was statistically significant (*Z* = −5.893, *P* = .000; Table 2).

**Table 2:** Comparison of fundus clarity and operation-related indicators between the 2 groups.

Group	Number of cases	Operation time (min)	PPV time (min)	Fundus clarity (scores)		Combined surgery, (n [%])	Corneal irrigation frequency (mean ± SD)
				4	3		
MSCL	21	87.10 ± 21.04	75.62 ± 21.81	21	0	10 (47.6%)	2.48 ± 0.87
BSS	25	72 (62, 94.5)	61 (48.5, 72.5)	9	16	17 (68.0%)	81.96 ± 24.86
Statistic value		−1.125*	−1.610*	20.608†		1.955†	−5.893*
*P*		.261	.107	.000		.162	.000 < .001

MSCL = modified soft contact lens, BSS = balanced salt solution, PPV = pars plana vitrectomy, SD = standard deviation.

* Mann–Whitney test.

† Chi-square test.

### 3.3. Comparison of the surgery view among different groups

**Figure 1.**
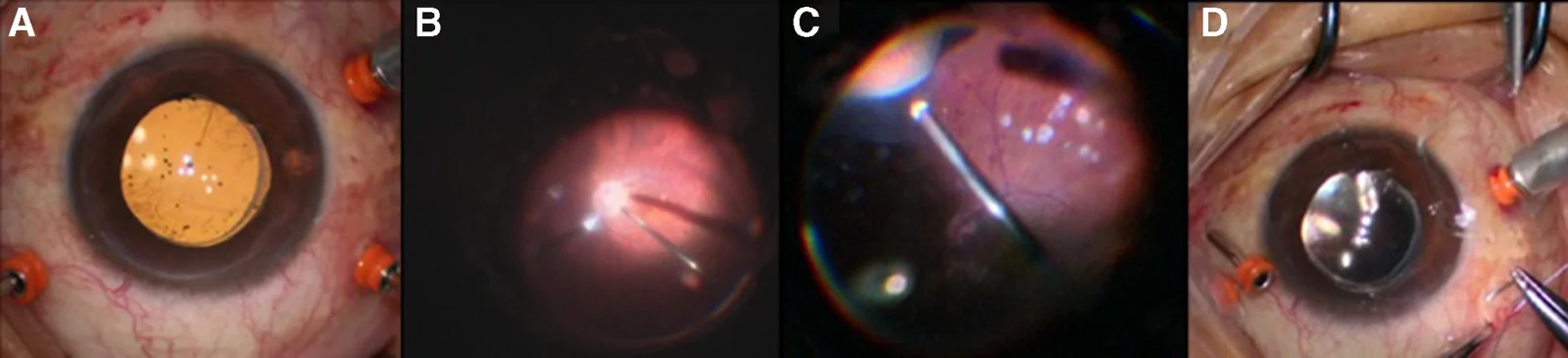
Images showing clarity of the surgical field during operation. (A) At the beginning of the procedure, the MSCL was placed on the surface of the cornea, and the white arrow indicates that a corneal bandage lens was placed on top. (B) The peripheral vitreous field remained clear throughout the operation. (C) The visual field remained clear and the object image was not deformed when the peripheral vitreous body was removed through scleral pressure. (D) When the MSCL was removed at the end of the operation, the cornea remained transparent as before. MSCL = modified soft contact lens.

As shown in Figure 1, the modified corneal bandage lens was placed on the cornea to ensure clear surgical vision at the beginning of the procedure. The midperipheral vitreous was clearly visible and excised, while the peripheral vitreous was effectively removed with external scleral top pressure and remained distinctly visible. All patients in the MSCL group attained level 4 (Fig. 1). By contrast, within the BSS liquid group, only 9 cases (36.0%) reached level 4 with poor visual field clarity, whereas 16 cases (64.0%) reached level 3, indicating statistically significant differences (χ^2^ = 20.608, *P* < .01; Table 2).

### 3.4. Corneal edema among different groups

The presence of corneal edema was absent in both groups prior to the surgical procedure. One day post-surgery, 5 patients in the MSCL group and 7 patients in the BSS group exhibited grade 1 corneal edema. Additionally, 1 patient from each group experienced grade 2 corneal edema on the first day after surgery. Both groups had 1 case of grade 1 corneal edema at 1 week following the operation. The Mann–Whitney *U* test conducted at 1 day and 1 week post-operation revealed no significant difference between the 2 groups (*Z* = −0.220, *P* = .826; *Z* = −0.125, *P* = .901). No instances of corneal edema were observed at 1 month after surgery (Table 3).

**Table 3:** Comparison of corneal edema degree before and after operation between the 2 groups.

Time	0 grade		1 grade		2 grade		3 grade		4 grade		Mann–Whitney *U*	*P* *
	MSCL	BSS	MSCL	BSS	MSCL	BSS	MSCL	BSS	MSCL	BSS		
Pre-op	21 (100%)	25 (100%)	0	0	0	0	0	0	0	0		
Post-1d	15 (71.4%)	17 (68%)	5 (23.8%)	7 (28.0%)	1 (4.8%)	1 (4.0%)	0	0	0	0	−0.22	.826
Post-1wk	20 (95.2%)	24 (96%)	1 (4.8%)	1 (4.0%)	0	0	0	0	0	0	−0.125	.901
Post-1mo	21 (100%)	25 (100%)	0	0	0	0	0	0	0	0		

MSCL = modified soft contact lens, BSS = balanced salt solution.

* Mann–Whitney test.

### 3.5. Comparison of central corneal thickness at different time points

In the MSCL group, there was a significant increase in central corneal thickness on 1 day post-op compared with pre-op. There was a significant decrease in central corneal thickness 1 week post-op compared with 1 day post-op, and no statistically significant difference in central corneal thickness recovery to preoperative levels at 1 month post-op. The BSS group also exhibited similar trends of changes (Table 4).

**Table 4:** Comparison of corneal thickness measured by AS-OCT at different time points before and after surgery between the 2 groups.

Groups	Pre-operation	Post-1d	Post-1wk	Post-1mo
MSCL	521.00 ± 25.38	558.19 ± 40.56	533.29 ± 28.43	524.71 ± 27.10
BSS	519.88 ± 31.41	570.96 ± 59.14	537.96 ± 37.78	523.28 ± 30.91
*F* *	0.017	0.701	0.217	0.027
*P*	.896	.407	.643	.869
Partial Eta square	0.000	0.016	0.005	0.001

AS-OCT = anterior segment optical coherence tomography, MSCL = modified soft contact lens, BSS = balanced salt solution.

* Repeated-measures, two-factor *F* test.

### 3.6. Comparison of corneal fluorescence staining scores

The Mann–Whitney *U* test showed that there was no statistically significant difference before operation, but there was a statistically significant difference at 1 day and 1 week after operation, and there was no statistically significant difference at 1 month after operation. In the MSCL group, the different time points were statistically significantly different (Friedman test = 10.664, *P* = .014). In the BSS group, at different time points, and the difference was statistically significant (Friedman test = 50.773, *P* = .000; Table 5). The Wilcoxon test was used to test between any 2 groups (Table 6): there was no statistically significant difference in corneal fluorescence staining between 1 week after surgery and before surgery in the MSCL group, while there was no statistically significant difference between 1 month after surgery and before surgery in the BSS group.

**Table 5:** Comparison of corneal fluorescence staining at different time points before and after surgery in the 2 groups.

Group	Pre-operation	Post-1d	Post-1wk	Post-1mo
MSCL	0.0 (0.0, 0.0)	1.0 (0.0, 2.0)	0.0 (0.0, 1.0)	0.0 (0.0, 0.5)
BSS	0.0 (0.0, 0.0)	4.0 (2.0, 6.0)	2.0 (0.0, 2.5)	0.0 (0.0, 0.0)
*Z* *	−1.571	−3.175	−2.535	−0.968
*P*	.116	.001	.011	.333

MSCL = modified soft contact lens, BSS = balanced salt solution.

* Mann–Whitney test.

**Table 6:** Comparison of corneal fluorescence staining at different time points with that before surgery.

Group	Post-1d		Post-1wk		Post-1mo	
	*Z* *	*P*	*Z* *	*P*	*Z* *	*P*
MSCL	−2.373	.018	−0.464	.642	−0.480	.631
BSS	−4.055	.000	−3.658	.000	−1.604	.109

MSCL = modified soft contact lens, BSS = balanced salt solution.

* Wilcoxon test.

### 3.7. Comparison of the breakup time at different time points

The BUT did not show significant changes in the MSCL group before or at different intervals (i.e., 1 day, 1 week, and 1 month) following surgery. However, in the BSS group, there was a significant decrease in BUT at 1 day post-op compared with pre-op. Additionally, 1 week post-surgery showed a significant increase in BUT compared with 1 day post-op, which eventually returned to preoperative levels by 1 month post-surgery (Table 7).

**Table 7:** Comparison of BUT between the 2 groups at different time points before and after surgery (mean ± SD).

Group	Pre-operation	Post-1d	Post-1wk	Post-1mo
MSCL	5.81 ± 1.54	5.71 ± 1.10	5.76 ± 1.58	5.71 ± 1.49
BSS	5.76 ± 2.15	3.32 ± 1.22	4.56 ± 1.56	5.32 ± 1.52
*F* *	0.008	48.20	6.719	0.783
*P*	.930	.000	.013	.381
Partial Eta square	0.000	0.523	0.132	0.017

MSCL = modified soft contact lens, BSS = balanced salt solution, BUT = tear film breakup time, SD = standard deviation.

* Repeated-measures, two-factor *F* test.

### 3.8. Comparison of OSDI scores between the 2 groups

The OSDI scores of the MSCL group at pre-op and 1 month post-op were 31.75 ± 10.12 and 32.54 ± 7.95, respectively, with no statistically significant difference (*T* = −0.316, *P* = .756). The OSDI scores of the BSS group at pre-op and 1 month post-op were 32.16 ± 10.91 and 34.33 ± 8.34, respectively, with no statistically significant difference (*T* = −1.499, *P* = .147). There was no significant difference between the 2 groups at the time points of the pre-op and 1-month post-op (*T* = −0.133, *P* = .895, *T* = −0.741; *P* = .463).

## 4. Discussion

In this study, we conducted a comprehensive comparison between MSCL and conventional BSS in vitreoretinal surgery. Our results demonstrate that the MSCL offers multiple advantages: it maintains consistent visual field clarity throughout surgery and eliminates the need for repetitive corneal irrigation with BSS, thereby facilitating rapid restoration of corneal epithelial barrier function and preservation of ocular surface homeostasis.

Vitreous surgery is commonly required for complex ocular conditions such as diabetic retinopathy, proliferative vitreoretinopathy, and ocular trauma. Given the prolonged duration and intricate manipulations involved in these procedures, maintaining a persistently clear surgical field is essential for achieving optimal outcomes. Previous studies have confirmed that cataract surgery, vitreous surgery, and other ophthalmic procedures can induce corneal epithelial damage, including punctate loss or large epithelial defects.^[13,14]^ Patients with preexisting corneal epithelial dysfunction, such as those with diabetes or dry eye, are particularly vulnerable to intraoperative injury and demonstrate delayed postoperative recovery.^[15–17]^ In a study of 255 patients undergoing PPV, corneal epithelial defects were observed in 23.1% of eyes on the first postoperative day, and diabetes and longer surgical duration were associated with a higher risk.^[15]^ Furthermore, persistent corneal epithelial defects occurred in 4.6% of cases following vitrectomy.^[16]^ These findings underscore the importance of protecting corneal integrity during surgery, which constitutes a primary objective of the present study.

Noncontact wide-angle viewing systems have become increasingly favored in vitreoretinal surgery because of their avoidance of direct corneal contact. However, the cornea remains exposed to air, making prolonged maintenance of a clear visual field challenging. Consequently, corneal wetting has emerged as a critical focus when using noncontact lenses. While BSS is the most commonly employed and cost-effective wetting agent, it has several limitations, including rapid evaporation necessitating frequent irrigation, patient discomfort under local anesthesia, and water accumulation in deep-set eyes, leading to blurred vision.

Alternative strategies, such as commercially available corneal protective agents and rigid gas-permeable contact lenses, have also been explored.^[4]^ Studies demonstrate that agents such as Provisc Ophthalmic Viscosurgical Device (ProVisc) and Ophthalmic Viscosurgical Device (DisCoVisc) require fewer intraoperative applications and provide longer maintenance of corneal moisture compared with BSS. However, their surface irregularities may compromise fundus visibility, particularly in peripheral vitreous regions, limiting their utility in delicate macular procedures. In 2018, a rigid polymethyl methacrylate contact lens was evaluated in 81 eyes, comparing rigid contact lens (RCL), corneal protective agent, and BSS groups.^[18]^ RCL provided a slightly expanded visible fundus area, and postoperative fluorescein staining scores were lower than in the BSS group. Despite these benefits, RCLs are difficult to disinfect repeatedly, prone to sliding, costly, and unsuitable for large-scale adoption. Furthermore, extended PPV procedures can still induce corneal edema with RCLs.

BSCLs, fabricated from silicone hydrogel, have been widely utilized for postoperative corneal healing and drug retention. Their oxygen permeability promotes epithelialization and reduces postoperative pain, while their scaffold-like structure facilitates uniform distribution of tear fluid across the ocular surface.^[19–21]^ BSCLs have demonstrated efficacy in corneal recovery following various ophthalmic procedures, including refractive surgery, corneal cross-linking, and vitreous surgery.^[21]^ Based on these advantages, we explored BSCL application during vitrectomy. However, commercially available BSCLs (13.8–14.0 mm diameter) often exceed corneal size, resulting in poor adhesion, lens sliding, entry of bubbles beneath the lens, and distorted visualization during peripheral vitreous maneuvers or scleral depression. These issues are further exacerbated in patients with smaller palpebral fissures or shallow conjunctival sacs, such as elderly Chinese patients.

Considering the strengths and limitations of existing approaches, we modified the BSCL to a diameter of 10.5 mm to ensure close corneal adherence without distortion, incorporating a siphon design to maintain corneal moisture and prevent rapid evaporation. This modified lens is cost-effective and well-suited for routine clinical use. Our study confirms that this modification provides a clear and enduring surgical field, reduces the need for frequent corneal irrigation, and enhances surgical efficiency and patient comfort.

Nevertheless, this study has certain limitations. The modified BSCL was not industrially mass-produced during the study period, limiting the availability of homogeneous lenses for broader clinical trials. Additionally, the precise mechanisms underlying corneal protection by the BSCL remain to be elucidated. Future investigations, including animal experiments and mechanistic studies, are warranted to further explore the protective effects of BSCL on the corneal epithelium.

## 5. Conclusion

The MSCL provides a clear and stable surgical field during vitreoretinal surgery, effectively reducing the need for repeated corneal irrigation and protecting corneal epithelial integrity. This approach enhances surgical efficiency and patient comfort, offering a practical and cost-effective strategy for routine clinical application. Further studies are warranted to elucidate the underlying mechanisms of corneal protection and to validate these findings in larger patient cohorts.

## Author contributions

**Conceptualization:** Jintao Xia, Xiaogang Yang.

**Funding acquisition:** Xiaogang Yang.

**Methodology:** Hongbing Zhang, Lina Cheng.

**Data curation:** Jintao Xia.

**Formal analysis:** Jintao Xia, Lina Cheng.

**Investigation:** Hongbing Zhang.

**Resources:** Hongbing Zhang.

**Software:** Jintao Xia, Hongbing Zhang.

**Writing – original draft:** Jintao Xia.

**Writing – review & editing:** Xiaogang Yang.
